# The deterrent effects of individual monoterpene odours on the dietary decisions of African elephants

**DOI:** 10.1007/s10071-023-01755-4

**Published:** 2023-02-17

**Authors:** Twané Bester, Melissa H. Schmitt, Adrian M. Shrader

**Affiliations:** 1 Mammal Research Institute, Department of Zoology and Entomology, University of Pretoria, Private Bag X28, Pretoria, 0028 South Africa; 2 School of Biology and Environmental Sciences, University of Mpumalanga, Private Bag X11283, Nelspruit, 1200 South Africa; 3 Department of Ecology, Evolution and Marine Biology, University of California, Santa Barbara, CA 93106 USA

**Keywords:** Pre-ingestive cues, Herbivory, Olfaction, Volatile organic compounds, Food preference, Foraging

## Abstract

African savanna elephants use pre-ingestive olfactory cues when making dietary choices, and previous research has observed that elephant diet choice is negatively correlated with vegetation species that contain high concentrations of monoterpenes. However, the frequency and concentration of monoterpenes can vary dramatically across plant species. Thus, we aimed to explore the effects that the odours of individual monoterpenes have on elephant diet choice and how these effects vary with concentration. To do this, we conducted three odour-based choice experiments focusing on eight common monoterpenes found in the woody plants in Southern African savannas. In the first experiment, we tested whether elephant diet choice for a frequently consumed plant (*Euclea crispa*) was influenced by the addition of the odour of an individual monoterpene at a set concentration. In the second experiment, we explored the relative deterrence of each monoterpene. Lastly, we tested how elephant diet choice varied as a function of the addition of individual monoterpene odours at 5%, 10%, and 20% concentrations. We found that the elephants avoided most individual monoterpenes at high concentrations, with the exception being α-pinene. Furthermore, we found that the odours of some individual monoterpenes were, in fact, more deterrent than others. In the third experiment, we found that the elephants avoided β-pinene, limonene, ocimene, γ-terpinene, and terpinolene across all concentrations, but only avoided sabinene and linalool at high concentrations. Ultimately, our results show that the odour of individual monoterpenes may deter elephant consumption, but that this deterrent effect depends on both the monoterpene and its concentration.

## Introduction

As a defence against herbivory, plants can produce chemical compounds via secondary metabolic processes that, when ingested, may have detrimental effects on herbivores (Fraenkel 1959; Herms and Mattson 1992; Iason 2005; Metlen et al. 2009). Plant secondary metabolites (PSMs) can decrease herbivore fitness by acting as antinutritional compounds that reduce the nutritive value of food as well as toxins that damage or hamper the productivity of the cells, tissues, and organs of herbivores (Estell 2010). Mammalian herbivores have evolved behavioural and physiological strategies to avoid the negative effects of PSMs (Iason 2005; Kohl and Dearing 2011). Physiological mechanisms include producing tannin-binding salivary proteins, regulating the amount of PSMs absorbed in the gut via permeability glycoproteins, and the biotransformation of PSMs into less harmful metabolites (McLean and Duncan 2006; Sorensen and Dearing 2006; Ward et al. 2020; Schmitt et al. 2020a, 2020b). Alternatively, behavioural strategies include avoidance, temporarily suspending consumption of foods after a certain amount has been eaten, and limiting the continuous ingestion of plants with high PSM content by increasing diet breadth (Freeland and Janzen 1974; Belovsky and Schmitz 1994; Provenza 1996; Boyle et al. 2005).

According to the detoxification limitation hypothesis, the diet breadth of herbivores is determined by their ability to metabolise PSMs (Freeland and Janzen 1974). This hypothesis asserts that, compared to specialists, generalist herbivores are physiologically less equipped to metabolise high levels of singular PSMs and have to employ behavioural strategies such as dietary mixing (Freeland and Janzen 1974; Provenza 1996). In doing so, the animal makes diet choices that include a variety of different plant species in their diet in an effort to avoid ingesting harmful amounts of any single PSM. Thus, plant–herbivore interactions are partly mediated by herbivores’ ability to recognise the risk of ingesting PSMs and make their diet choices accordingly (Provenza 1996).

Mammalian herbivores can base their diet choices on pre-ingestive cues and post-ingestive feedback (Provenza 1996; McCrickerd and Forde 2015; Finnerty et al. 2017). Post-ingestive feedback consists of the physiological effects following the consumption of different foods, which, in the case of foods with excessive amounts of PSMs, includes nausea and malaise (a general feeling of unwellness; Provenza 1995). Pre-ingestive cues consist of plants’ visual, gustatory, and olfactory characteristics that mammalian herbivores detect as they move across the landscape (see olfactory landscape; Finnerty et al. 2022) and utilise to differentiate between food items (Iason 2005; Lev-Yadun and Gould 2007; Kohl and Dearing 2011; Stutz et al. 2017). In particular, plant odour has been demonstrated to be a crucial pre-ingestive cue for some mammalian herbivores (e.g., Bedoya-Pérez et al. 2014; Stutz et al. 2017; Schmitt et al. 2018). Volatile organic compounds (VOCs) are odoriferous PSMs that frequently serve as olfactory cues to mammalian herbivores (Elliott and Loudon 1987; Stutz et al. 2016; Finnerty et al. 2017; McArthur et al. 2019). A key group of VOCs that affects mammalian herbivory is monoterpenes (Elliott and Loudon 1987; Seigler 1998). Monoterpenes are present in a wide range of plants and play a large role in herbivore-plant interactions by generally acting as a feeding deterrent (Lawler et al. 2000; Dziba and Provenza 2008; Champagne et al. 2019). Monoterpenes can also incur post-ingestive costs and may exhibit toxic effects on the organs of herbivores (Sperling 1969; Acamovic and Brooker 2005). Thus, mammalian herbivores may avoid plants containing high concentrations of monoterpenes due to the potential health risks of ingesting these defence compounds (i.e., direct defence; Boyle et al. 1999; Vourc’h et al. 2002; Ramak et al. 2014). Alternatively, monoterpenes could serve as an indirect defence against herbivory by, for example, providing odour refuges for preferred plants or being associated with the presence of another, potentially more harmful, PSM (Lawler et al. 1998; Moore et al. 2004; Massei et al. 2007; Dicke and Baldwin 2010; Bedoya-Pérez et al. 2014).

The African savanna elephant (*Loxodonta africana*) is a mammalian herbivore that uses odour as a pre-ingestive cue to make both within and between-patch foraging decisions (Schmitt et al. 2018; Nevo et al. 2020). Using odour as a pre-ingestive cue is particularly useful for African elephants because they have high olfactory acuity (Schulte and LaDue 2021) and are native to sub-Saharan environments that often contain heterogeneously distributed resources (De Knegt et al. 2008). Additionally, using their keen sense of smell to guide their selective foraging behaviour could drastically decrease the time elephants spend searching for palatable foods (Stutz et al. 2017; Orlando et al. 2020). Elephants are generalist, mixed-feeders, and the proportion of grasses and woody plants within their diets vary geographically (Koch et al. 1995; Codron et al. 2006). Some populations primarily browse, whereas others primarily graze (Williamson 1975; Shrader et al. 2012). Moreover, the diet of African elephants also differs temporally (Barnes 1982), with many populations of elephants shifting to feed on woody plants during the dry season (Van Der Merwe et al. 1990; Codron et al. 2006; Owen-Smith and Chafota 2012) and grasses during the wet season (De Boer et al. 2000). Unlike grasses, which generally contain few PSMs, woody plants are often chemically defended by both non-volatile (e.g., tannins) and volatile compounds (e.g., terpenes; Rhoades and Cates 1976; Bryant et al. 1991). Thus, by browsing on woody plants, elephants are at risk of experiencing the deleterious effects of PSMs. Previous studies have found that elephant diet choice is negatively correlated with vegetation species that contain high concentrations of monoterpenes (Schmitt et al. 2020c). However, the role that specific monoterpenes and their concentrations play in determining elephant diet choice is unknown.

Monoterpenes are a chemically diverse class of terpenes that may have varying physiological implications (Seigler 1998; Acamovic and Brooker 2005; Iason 2005), and elephants may consequentially display different degrees of avoidance towards individual monoterpenes (Freeland and Janzen 1974; Forbey et al. 2011; Ulappa et al. 2014). Additionally, mammalian herbivores have different toxin-thresholds for individual monoterpenes (Boyle et al. 2005; Nobler et al. 2018; Marschner et al. 2019), which could dictate the concentration at which herbivores avoid them (Wiggins et al. 2006). Ultimately, the influence that different individual monoterpenes have on elephant diet choice remains unclear.

To explore this knowledge gap, we ran three odour-based choice experiments where we used a population-level approach to test (1) whether elephant diet choice was influenced by the addition of the odour of an individual monoterpene at a set, high concentration, (2) the relative deterrence of each monoterpene, and (3) how elephant diet choice varied as a function of the addition of individual monoterpene odours at a range of concentrations. The aim of these three experiments were to determine whether the odours of monoterpenes deter elephants at high concentrations, whether deterrence differs between individual monoterpenes, and whether the deterrence of an individual monoterpene diminishes at lower concentrations. To do this, we used individual monoterpenes found in varying concentrations and occurrences in the elephants’ preferred and avoided woody plants in the study area (see Schmitt et al. 2020c). Given that monoterpene concentration is negatively correlated with elephant consumption (Schmitt et al. 2020c), we predicted that the elephants would avoid the odour of monoterpenes at high concentrations. Furthermore, similar to other herbivores, elephants likely have varying physiological capacities to detoxify different individual monoterpenes (Boyle et al. 2005; Nobler et al. 2018; Marschner et al. 2019). As such, we expected the elephants to prefer or avoid certain monoterpenes when compared to others. Lastly, increasing the concentration could also increase the likelihood of the monoterpenes overwhelming the elephants’ detoxification pathways and inducing negative post-ingestive feedback (Freeland and Janzen 1974; Dearing et al. 2000; Sorensen et al. 2004). Thus, we predicted that the elephants would be more likely to avoid monoterpenes at high added levels than low added levels.

## Methods

### Sampling

We conducted our study during August and October 2019, and September and October 2020 at the Adventures with Elephants facility in Bela-Bela, Limpopo, South Africa (24°46′53.8″S, 27°57′03.3″E). To test the influence that individual monoterpenes have on elephant diet choice, we ran odour-based choice experiments on five semi-tame adult African elephants. The group of elephants consisted of two males (Chova 24 and Chishuru 22 years of age) and three females (Nuanedi 18 years, Mussina 18 years, Shan 19 years). The elephants were initially acquired from the wild between 2007–2008 from Greater Kuduland Safaris, Soutpansberg, Limpopo, South Africa (22°36′15.0"S, 30°10′25.2"E) and Farm Grootgeluk, Soutpansberg, Limpopo, South Africa (24°31′0.1″S, 28°43′0.1″E). In 2010, all five elephants were relocated to the Adventures with Elephants facility from the Elephants for Africa Forever Facility, Mooketsi, Limpopo, South Africa (23°28′22.5″S 30°06′48.2″E). Prior to their acquisition in 2008, the elephants roamed and fed freely across the landscape, but then followed a similar feeding regime at the Elephants for Africa Forever Facility and Adventures with Elephants facility (i.e., roaming in the bush during the day with occasional dietary supplements of nutritional grasses). We gathered data during three experimental sessions at 8h30, 12h00, and 15h00. During these sessions, each elephant was presented with a choice combination and would participate in five odour-based choice trials per combination (see details below). The first experiment consisted of eight choice combinations (i.e., 40 choice trials per elephant). Experiment 2 consisted of 21 choice combinations (i.e., 105 choice trials per elephant), and experiment 3 consisted of 32 choice combinations (i.e., 160 choice trials per elephant). The number of trials any individual elephant faced per day ranged between 5 and 15 (e.g., 5 trials in the morning, 5 in the afternoon, and 5 in the late afternoon), with 10 being the mean daily number of trials. For the experiments, we used eight monoterpenes that were found in the natural woody vegetation eaten by the elephants at the Adventures with Elephants facility (Schmitt et al. 2020c). The physiological effects that monoterpene consumption has on elephants are unknown, but these compounds are still likely toxic when consumed in high quantities. As such, the experimental procedure was specifically designed to ensure that the elephants would not ingest any of the monoterpenes. Additionally, all experiments were conducted by the elephants’ personal handlers to ensure the safety of all participating parties as well as the comfort of the elephants.

### Odour-based choice trials

**Fig. 1:**
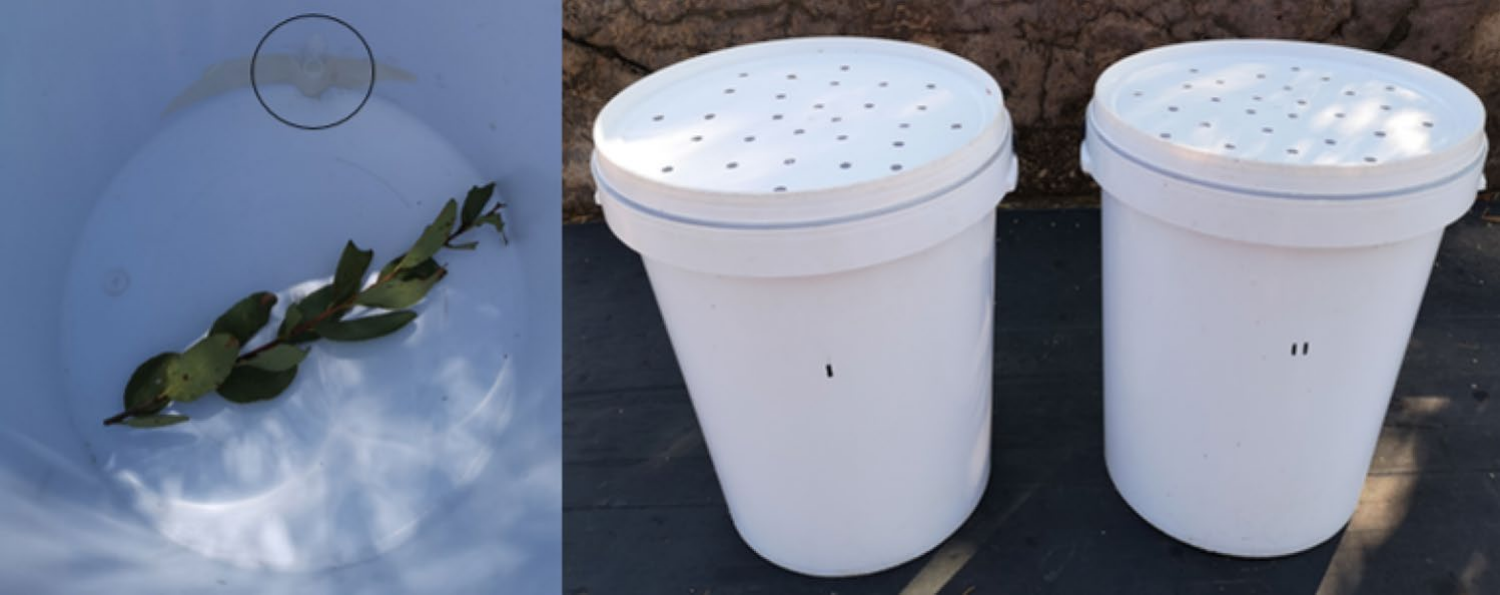
Bucket with a branch of *E. crispa* (dry weight = ca. 5 g) inside and a 2 mL Eppendorf tubule (circled in black) adhered near the bottom (Left). The two buckets with perforated lids standing side-by-side (Right)

The odour-based choice experiments consisted of trials where the elephants used olfaction to choose between two 25 l opaque plastic buckets (height: 440 mm, top diameter: 300 mm, bottom diameter: 255 mm) with perforated lids (28 holes separated by ca. 3 cm; Fig. 1). The purpose of the lids was to ensure that the elephants could obtain olfactory, but not visual, information on the content of the buckets. Each bucket contained a small branch of *Euclea crispa* (dry weight = ca. 5 g) as a food reward during the trials (Fig. 1). We used *E. crispa* because it is a woody plant that is frequently encountered and consumed by the elephants (Schmitt et al. 2018). Each bucket also contained a 2 mL Eppendorf tubule that we adhered inside near the bottom of the bucket (Fig. 1). In the experimental bucket, the Eppendorf tubule contained an individual monoterpene suspended in the largely odourless liquid, dipropylene glycol. As a control, we attached in the second bucket an Eppendorf tubule that only contained dipropylene glycol. All eight monoterpenes, including α-pinene: 98% purity, (‒)-β-pinene: 99% purity, linalool: 97% purity, (R)-( +)-limonene: 97% purity, ocimene: ≥ 90% purity, sabinene: 75% purity, terpinolene: ≥ 95% purity, and γ-terpinene: 97% purity, were produced by Sigma-Aldrich Inc.

**Fig. 2:**
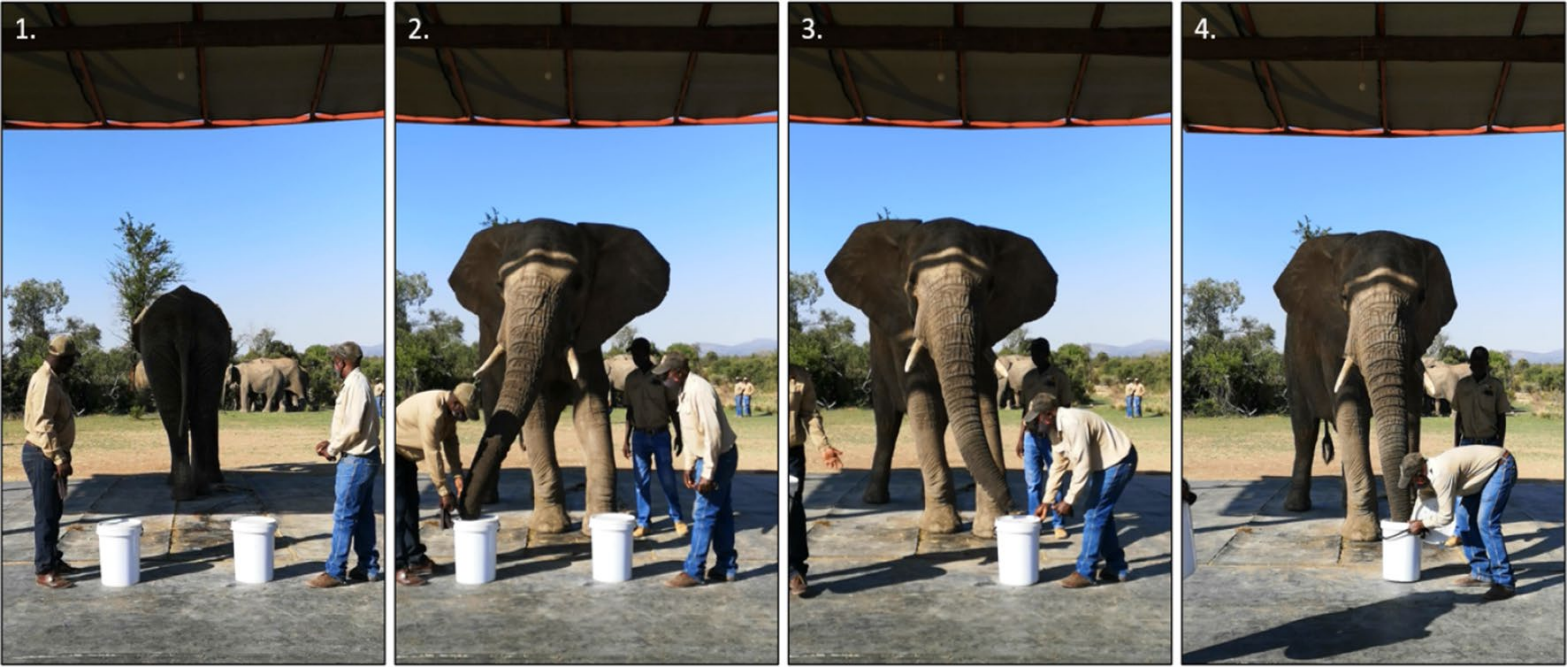
The smell–smell–choose procedure followed during the trials: 1. Buckets and handlers are positioned while the elephant faces away. 2. The elephant is instructed to turn around and smell both buckets. 3. The elephant indicates the preferred bucket by placing its trunk on the lid. 4. The unselected bucket is taken away, and the lid of the preferred bucket is removed so that the elephant has access to the *E. crispa* branch

As per Schmitt et al. (2018) and Wood et al. (2022), a smell–smell–choose procedure was followed during each trial. The trials started with the prepared buckets and the elephants’ handlers positioned roughly 5 m away from the elephant (Fig. 2). During this phase, the elephant was facing away from the buckets with a third handler by its side who issued instructions to the elephant and ensured its comfort. First, the elephant was instructed to turn, walk forward, and smell each of the buckets (i.e., smell-smell; Fig. 2). Once it had smelled both buckets, the elephant was then instructed to tuck its trunk back and then ‘choose’ which bucket it preferred by placing its trunk on the bucket’s lid. The unselected bucket was then promptly taken away, while the selected bucket’s lid was removed to grant the elephant access to the branch of vegetation inside (Fig. 2). The elephant would retrieve the branch and could then decide whether to consume it or not. This procedure constituted one trial, which was then repeated five times per session per elephant.

The position of the handlers and buckets were swapped randomly between each trial to ensure that the elephants’ decisions were based solely on the buckets’ odours and not the position of the bucket or the two handlers. The third handler issuing instructions to the elephant (i.e., the handler closest to the elephant) remained stationary between trials. However, the side of the elephant on which the third handler was located was random and their position would vary between elephants and sessions. All handlers remained blind to the monoterpene content of the buckets, and we limited the experimenter’s intervention during the trials to cleaning the buckets, replacing the *E. crispa* branch, and instructing the handlers to swap positions and buckets. We took these precautions to prevent the elephants from utilising non-verbal cues from the handlers and experimenter and to reduce human impact during the experiment (Chu et al. 2022).

To prevent the elephants from cueing off each other during the trials, we ensured a minimum distance of 8 m between the buckets and the other elephants that were not actively partaking in the trials. Between each trial, we wiped the insides and lids of both buckets with one of three damp cloths. After each session with an individual elephant, we rinsed the cloth thoroughly with water, laid it out in the sun, and swapped it out for another cloth before starting the next session. Additionally, we switched the lids of the buckets ca. every two trials. We took these measures to ensure that the mucous from the elephants’ trunks on the lids or inside the buckets did not affect their choices (i.e., provided an olfactory marker). We also scrubbed the lids after each session with water and coarse dirt to remove any remaining mucous. After completing the sessions with all five elephants, we washed the buckets and their lids with 95% ethanol and left them to dry in the sun for ca. 3 h before the next session. This was done to remove any lingering odours from the monoterpene solutions and elephant mucus from the previous session.

### Training

We trained the elephants for four days before they participated in the odour-based choice experiments. The goal of the training was to ensure that the elephants understood the basis of the experiment (i.e., choosing the bucket they preferred based on the content’s odour). The training consisted of trials where the elephants were presented with a choice between a bucket containing a branch of *E. crispa* (dry weight = ca. 5 g) and a bucket containing a branch of *Olea europaea* subsp. africana (dry weight = ca. 3 g). In general, elephants avoid *O. europaea*, but sometimes eat *E. crispa* (Schmitt et al. 2020c). Upon choosing, the elephants were allowed to retrieve the branch from the bucket they selected and decide whether to eat it. Thus, if the elephants chose the bucket that contained *E. crispa*, they made the choice that could provide a potential food reward (i.e., positive reinforcement). Yet, due to its unpalatable nature, choosing the bucket with *O. europaea* resulted in the elephants selecting something they do not eat (i.e., a “bad” choice) and thus, the selection resulted in negative reinforcement. However, they were not required to eat either branch, and sometimes chose not to. This procedure ensured that the elephants understood the “you get what you choose” reward system of the odour-based choice experiments, and that their choices were driven by both their relative preference for *E. crispa* as well as their avoidance of *O. europaea*. The elephants were considered successfully trained once they only chose the buckets containing *E. crispa* (i.e., all choices made consisted of the buckets containing *E. crispa*) for five consecutive trials spanning an entire day.

### Deterrence of individual monoterpene odours at high concentrations

To determine whether the odour of monoterpenes alone deter elephant diet choice, we ran odour-based choice trials where we presented the elephants with a choice between a control bucket and a bucket containing a 2 mL Eppendorf tubule with a monoterpene solution at 30% concentration. In the 30% solution, the amount of added monoterpenes totalled to 10.2 ± 0.26% of the dry weight (gDW) of the food reward (*E. crispa*, dry weight = ca. 5 g). Previous studies have considered this to be a very high level of added monoterpenes (Wiggins et al. 2003; Dziba and Provenza 2008; DeGabriel et al. 2009). However, unlike the aforementioned studies, the elephants did not consume the monoterpenes. Thus, we used a high level of added monoterpenes to first determine whether the elephants avoid the buckets despite the absence of added post-ingestive repercussions.

### Differences in deterrence of individual monoterpenes compared to each other

In our second experiment, we determined whether individual monoterpenes differ in their deterrence of elephant consumption by running odour-based choice trials where the elephants received a choice between two buckets that both contained a 2 mL Eppendorf tubule with a monoterpene solution at 30% concentration. However, the individual monoterpenes suspended in dipropylene glycol in these solutions differed between the two buckets. Due to circumstances, the monoterpenes tested in this experiment was limited to α-pinene, β-pinene, linalool, limonene, ocimene, terpinolene, and γ-terpinene.

### Deterrence of individual monoterpenes at decreasing concentrations

In our third experiment, we ran odour-based choice trials similar to our first experiment, but with the eight individual monoterpenes at lower concentration. In these trials, the 2 mL Eppendorf tubules adhered inside the buckets contained monoterpene solutions at 5%, 10%, and 20% concentrations. The total amount of monoterpenes in the three solutions were 1.7 ± 0.04%, 3.4 ± 0.09%, and 6.8 ± 0.17% DW of the food reward, respectively. The concentrations that we tested were considered to range from very low to medium added levels (Wiggins et al. 2003; Dziba and Provenza 2008; DeGabriel et al. 2009). We then compared the avoidance that the elephants showed towards the individual monoterpene at 5%, 10%, 20%, and 30% concentration to determine whether concentration influences the deterrent effect that monoterpenes have on elephant diet choice.

### Data analysis

The data gathered from the odour-based choice experiments consisted of series of binary choices made by the elephants (i.e., choices between two buckets). The individual elephants were each tested five times per combination of choices. Due to our limited sample size, our data did not allow us to generate meaningful results on variation in proportion of choices made by the elephants at an individual level. As such, we treated the data gathered in these experiments as repeated measures and used generalized estimating equations (GEEs), which are semiparametric repeated measures analyses that are appropriate to explore average, population-level responses of subjects and can handle small sample sizes (Ma et al. 2012; Naseri et al. 2016). We defined the subject variable as the individual elephants and the within-subject variable as the combination of choices presented to the elephants.

We used GEEs because they allow analysis for repeated measures and compensate for non-independence in the data (Ballinger 2004). In the case of this study, non-independence may have arisen from the elephants remembering their choices in the previous trials. GEEs use a population-level approach based on a log quasi-likelihood function and deliver population-averaged parameter estimates. In GEEs, coefficients are marginal effects that are measured at the population level and average across all subjects in the data (Schluchter 2008). The goodness of fit measures in GEEs are displayed as Quasi Likelihood under Independence Model Criterion (QIC), which allowed us to choose the best working correlation function.

The model incorporated an exchangeable working correlation matrix and a binomial error distribution with a logit link function. In this study, the GEEs modelled the proportion of elephants making a certain choice given the coefficients and compared this proportion to the 50% distribution expected under random selection. The data were then back-transformed from the logit-scale for graphical representation. We used the means and asymmetrical 95% confidence intervals (CIs) to determine whether elephants avoided or randomly selected (i.e., 50% distribution) the individual monoterpenes.

To determine whether the odour of individual monoterpenes at high concentrations deter elephant consumption (experiment 1), we analysed the number of choices that elephants selected the control bucket with no added odour over the bucket containing the odour of an individual monoterpene at 30% concentration. In the second experiment, we determined whether individual monoterpenes had different deterrent effects by analysing the number of the choices where the elephants preferred certain monoterpenes when compared to others. Thirdly, we determined whether elephants avoided individual monoterpenes at varying concentrations and analysing the number of the choices where the elephants selected the control bucket with no added odour over the bucket containing an individual monoterpene at 5%, 10%, 20%, and 30% concentration. In experiment 1 and 3, we defined the reference variable as the selection of the control bucket and the response variable as the selection of the bucket containing the added odour of an individual monoterpene. Given that both buckets contained the odour of an individual monoterpene in experiment 2, we defined the response variable as the individual monoterpene that was chosen less frequently in comparison to the other monoterpene in the choice combination, which we defined as the reference variable.

To confirm that the choices made by the elephants did not stabilize across the course of the five trials, we explored how the average number of similar choices made by the elephants per choice combination varied across the five trials. We used a generalized linear model with a Poisson distribution and a log-link function with "number of choices" as the dependent variable and "trial" as the main effect. If the elephants’ choices became more stable over the trials, we expected the average number of similar choices to be significantly different across the trials and that the average number of similar choices would increase with trial number. However, we did not find this (*χ*^2^ = 4.811, df = 4, *p* < 0.307) and conclude that the stability of the elephants’ choices was similar across all trials.

Finally, we established whether the elephants avoided certain choices in the choice combinations by using the marginal means of the choices made by the elephants and their 95% CIs and comparing these values to the 50% distribution expected under random selection. Means and CIs above the 50% proportion threshold indicated that the elephants avoided one of the choices in the combination. Alternatively, CIs that overlapped with the 50% proportion threshold indicated random selection between the two choices. All statistical analyses were conducted using IBM SPSS version 26.0 (IBM Corp. 2019).

## Results

### Deterrence of individual monoterpene odours at high concentrations

**Fig. 3:**
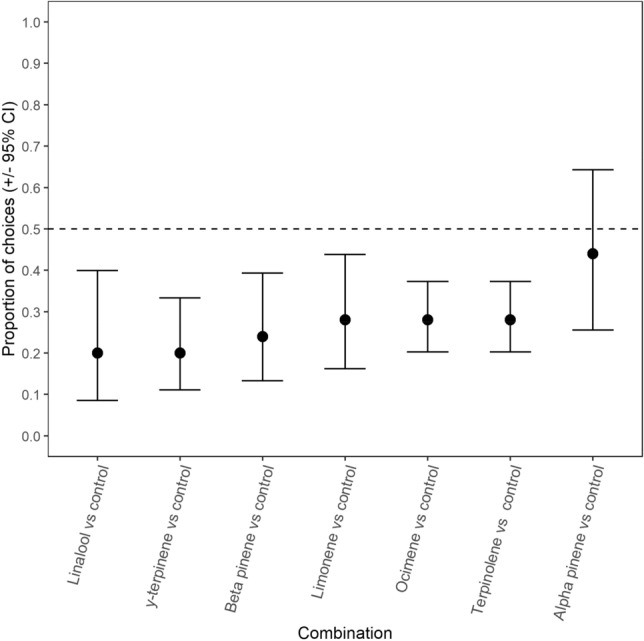
The proportion of choices where the elephants (± 95% confidence intervals) selected the bucket containing the odour of an individual monoterpene over the control bucket. Confidence intervals that overlap the 0.5 proportion line indicate no difference from random selection. Means and CI below the 0.5 proportion indicate avoidance of the specific monoterpene

When given a choice between the dipropylene glycol (control odour) and individual monoterpenes at 30% concentration, the elephants avoided most individual monoterpenes (Fig. 3; GEE: *χ*^2^ = 4.364, *p* = 0.359). Out of the eight monoterpenes, α-pinene was the only monoterpene that the elephants did not avoid at 30% concentration (Fig. 3).

### Differences in deterrence of individual monoterpenes compared to each other

**Fig. 4:**
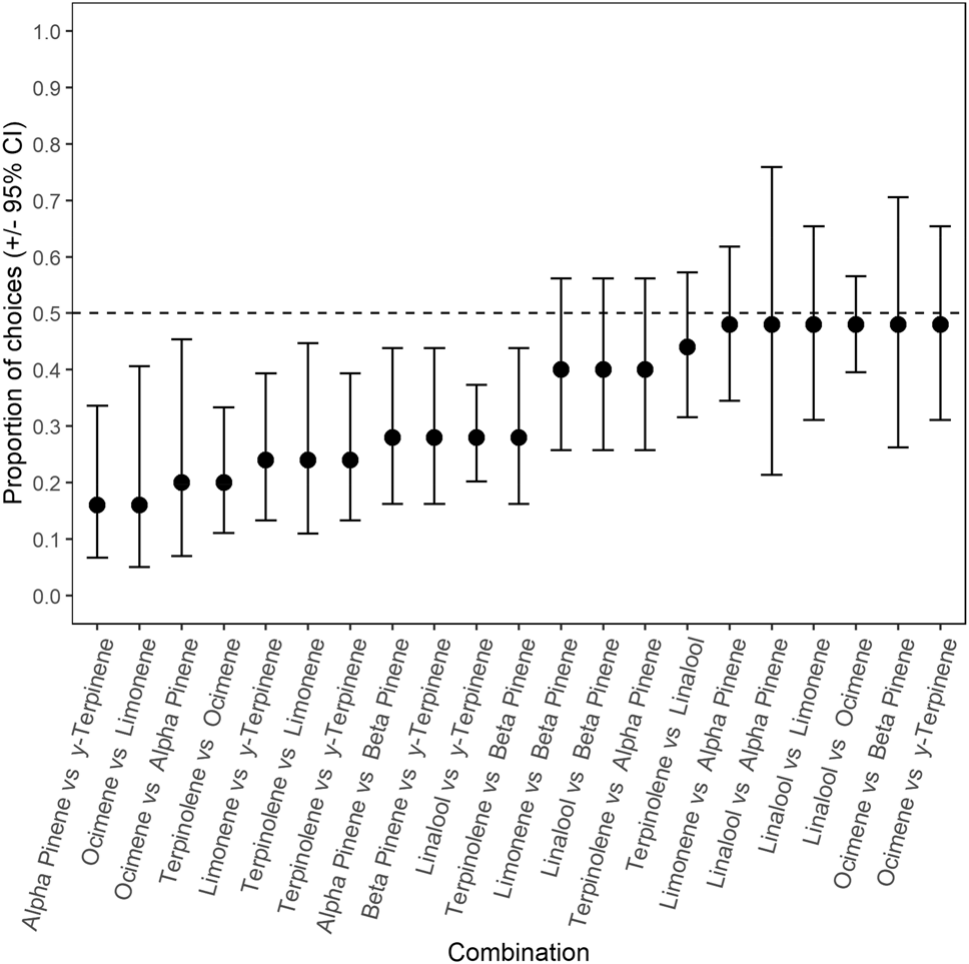
The proportion of choices where the elephants (± 95% confidence intervals) selected the bucket containing the odour of the monoterpene first listed in the x-axis when compared to the second listed monoterpene. Choice combinations are listed in increasing order of proportion of the elephants’ choices that consisted of the monoterpene first listed in the x-axis. Confidence intervals that overlap the 0.5 proportion line indicate no difference from random selection. Means and CI below the 0.5 proportion indicate avoidance of the monoterpene listed first on the x-axis

Analysis of the proportion of avoidance among all 21 combinations showed that there were significant differences in selection across monoterpene combinations (Fig. 4; GEE: *χ*^2^ = 64.062, *p* < 0.001). In 11 of the 21 combinations, the elephants showed significant avoidance for a particular monoterpene in the combination (Fig. 4). In the remaining 10 choice combinations, the elephants randomly selected between the monoterpenes offered to them. Thus, no individual monoterpene was universally avoided or preferred when compared to the other individual monoterpenes.

### Deterrence of individual monoterpenes at decreasing concentrations

**Fig. 5:**
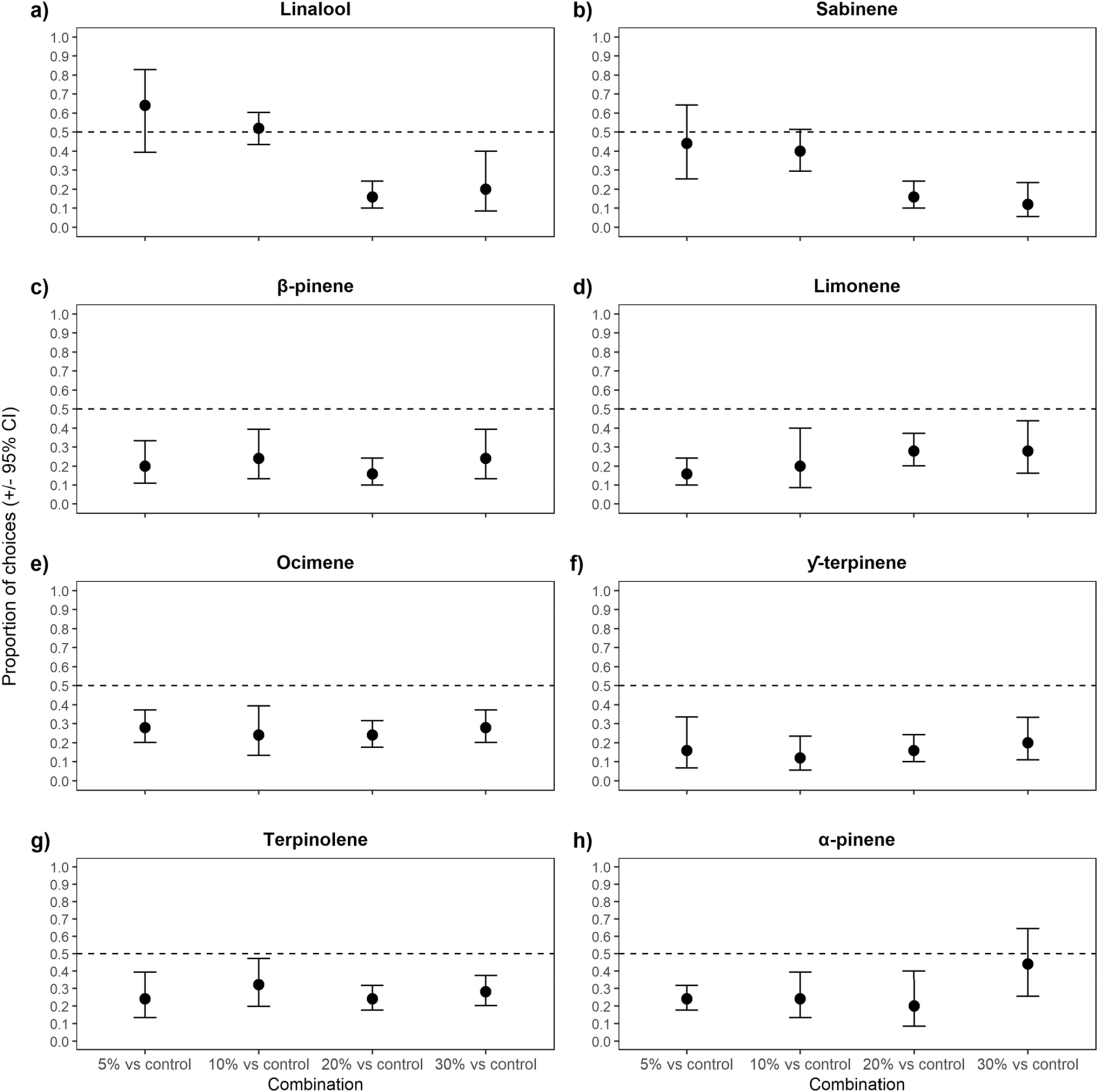
The proportion of choices where the elephants (± 95% confidence intervals) selected the bucket containing the monoterpene **a** linalool, **b** sabinene, **c** β-Pinene, **d** limonene, **e** ocimene, **f** γ-terpinene, **g** terpinolene, and **h** α-Pinene at 5%, 10%, 20%, and 30% concentration compared to control odour (Dipropylene glycol). Confidence intervals that overlap the 0.5 proportion line indicate no difference from random selection. Means and CI below the 0.5 proportion indicate avoidance of the individual monoterpene

When given a choice between the control odour (dipropylene glycol) and linalool, the elephants did not show a preference (i.e., random selection) at the 5% and 10% concentrations, but significantly avoided linalool at the 20% and 30% concentrations (Fig. 5a; GEE: *χ*^2^ = 96.526, *p* < 0.005). The elephants showed the same pattern when given a choice between sabinene and the control odour (Fig. 5b; GEE: *χ*^2^ = 406.143, *p* < 0.005). Thus, the proportion of avoidance for linalool and sabinene increased at 20% and 30% concentration (Fig. 5a, b).

The monoterpenes that were avoided across all concentrations included β-pinene (Fig. 5c, GEE: *χ*^2^ = 65.765, *p* < 0.005), limonene (Fig. 5d, GEE: *χ*^2^ = 5.066, *p* = 0.176), ocimene (Fig. 5e, GEE: *χ*^2^ = 1.307, *p* = 0.727), γ-terpinene (Fig. 5f, GEE: *χ*^2^ = 1.575, *p* = 0.665), and terpinolene (Fig. 5g, GEE: *χ*^2^ = 0.982, *p* = 0.806). Thus, the avoidance of these monoterpenes did not scale with concentration. However, α-pinene was not avoided across all concentrations (Fig. 5h; GEE: *χ*^2^ = 12.988, *p* = 0.005). Instead, when given a choice between the control odour and α-pinene, the elephants significantly avoided α-pinene at the 5%, 10%, and 20% concentrations but did not show a preference (i.e., random selection) at a 30% concentration. As such, avoidance did not scale with concentration for α-pinene.

### Variation in choices made by individual elephants

Overall, we did not observe any instances where the proportion of choices were predominantly driven by the choices made by an individual elephant. However, we did observe instances where some of the elephants displayed random selection and others clear preferences/avoidance for certain choices, which are represented by asymmetry in the confidence intervals in Figs. 3–5, but this dichotomy did not influence whether an option was considered avoided or not (i.e., cause the confidence intervals to overlap the 0.5 proportion line). Thus, although the elephants did not always unanimously avoid/prefer certain odours, the variation in the choices made by the elephants was not the primary driver for the population-level results obtained from the GEEs.

## Discussion

Our study suggests that the odour of monoterpenes can deter elephants from consuming certain plants, and that this deterrence differs between individual monoterpenes and their concentrations. Monoterpenes are plant secondary metabolites that provide pre-ingestive olfactory cues and may induce negative physiological consequences when consumed (Schwartz et al. 1980; Striby et al. 1987). Elephants use pre-ingestive cues to make dietary decisions and monoterpenes are relatively more abundant in the plants that they avoid compared to preferred plants (Schmitt et al. 2018, 2020c). A possible explanation for our results may be that the elephants have learned to associate the odour of monoterpenes with post-ingestive repercussions of these compounds themselves, or the plants in which they occur (direct or indirect defence; Provenza 1996; Lawler et al. 1998; Boyle et al. 1999; Moore et al. 2004). However, because the elephants did not consume the monoterpenes in this study, and because the total amount of monoterpenes within avoided plants are unknown, this explanation remains conjectural. Alternatively, the elephants may have based their dietary choices on inherited food aversions (Thorhallsdottir et al. 1990; Biquand and Biquand-Guyot 1992; Myers and Sclafani 2006).

As a direct defence against elephant herbivory, the deterrent effect that monoterpene odours have on elephant diet choice would primarily be attributed to the toxicity of these compounds (Boyle et al. 1999; Ramak et al. 2014). For example, terpinolene and α-terpineol exhibit cytotoxic effects on the liver, kidney, lungs, and neurological tissues by disrupting cell plasma membranes in mammals (Agus 2021). Similarly, liver and tissue damage caused by degeneration of membrane structures within the cells has been observed in mice after the administration of relatively high doses of 1,8-cineole (Xu et al. 2014). The post-ingestive costs and consequential negative post-ingestive feedback of consuming monoterpenes would likely only occur when consumed in quantities above the elephants’ toxin thresholds (Freeland and Janzen 1974; Estell 2010). In the first experiment, the elephants avoided seven out of the eight monoterpene odours at 30% concentration, (i.e., 10.2 ± 0.26% dry weight; Fig. 3). Thus, if monoterpenes act as a direct defence against elephant herbivory, the odour of β-pinene, linalool, limonene, ocimene, sabinene, terpinolene, and γ-terpinene at 30% concentration may be pungent enough to be associated with monoterpene levels that reach the elephants’ toxin threshold and, consequently, cause negative post-ingestive feedback when ingested (Freeland and Janzen 1974; Provenza 1996; Dearing and Cork 1999; Marschner et al. 2019). Furthermore, the elephants would likely be more inclined to avoid the pre-ingestive odour cues of monoterpenes that would incur greater post-ingestive costs when compared to the odour of a different individual monoterpene (Freeland and Janzen 1974; Provenza 1996). Thus, the results of experiment 2 would imply that monoterpenes elicit different physiological repercussions, seeing as, out of 21 choice combinations, the elephants showed clear avoidance of 11 monoterpene odours when directly compared to another monoterpene odour at 30% concentration (Fig. 4). This makes sense, seeing as herbivores display varying capacities to detoxify different individual monoterpenes (Nobler et al. 2018; Hernandez-Ortega et al. 2018).

Given that monoterpenes are less likely to overwhelm the detoxification pathways of herbivores when consumed at very low concentrations, we predicted that, if these compounds act as a direct defence against elephant herbivory, the elephants would not avoid the odour of the individual monoterpenes at very low concentrations (Freeland and Janzen 1974; Provenza 1996; Estell 2010). However, in experiment 3, we found that the elephants avoided most of the monoterpene odours across all concentrations (i.e., β-pinene, limonene, ocimene, terpinolene, and γ-terpinene; Fig. 5). Previous studies done on the dietary responses of two other generalist herbivores, namely cottontail rabbits (*Sylvilagus nuttalli*) and common brushtail possums (*Trichosurus vulpecula*), observed immediate reduction in food intake after a low concentration of the individual monoterpene, 1,8-cineole, was added (DeGabriel et al. 2009; Shipley et al. 2012). Thus, similar to these generalist herbivores, elephants may display fine-tuned dietary reactions towards increased concentrations of highly avoided monoterpenes observed via odour cues, which would also explain why they more frequently avoid plants that contain them (Freeland and Janzen 1974; Schmitt et al. 2020c). In contrast, the elephants did not avoid linalool and sabinene at 5% and 10% concentrations (1.7 ± 0.04% DW and 3.4 ± 0.09% DW, respectively; Fig. 5), Similar studies done on roe deer (*Capreolus capreolus*) and sheep also found that sabinene did not decrease food intake (Estell et al. 2000; Vourc’h et al. 2002), which may be indicative of the low bioactivity and, consequently, post-ingestive cost of this monoterpene. Furthermore, linalool is relatively more abundant in the odour profiles of preferred plants than avoided plants, which implies that it is frequently consumed by the elephants (Schmitt et al. 2020c). As such, if the pungency of monoterpene odours is correlated with the negative post-ingestive feedback of consuming these compounds at high concentrations, the elephants may physiologically be able to tolerate *E. crispa* that contains linalool and sabinene at low levels (Freeland and Janzen 1974; Marsh et al. 2006).

As a toxic direct defence compound, the post-ingestive costs monoterpenes and consequential deterrence of individual monoterpene odours likely rely on a variety of external factors. For example, because the elephants used in this study were semi-tame (i.e., released to forage in their natural habitat during the day) and the experimentation spanned over several days, it is possible that, prior to the trials, the elephants had consumed plants that already contained high levels of the monoterpenes that were being tested. The increased blood concentration of these monoterpenes could potentially cause the elephants to avoid their odours (Boyle et al. 2005). Consumption of specific individual monoterpenes prior to the experimental trials could explain why no individual monoterpene was universally more deterrent than the others in experiment 2 (Fig. 4), as well as why most individual monoterpene odours were avoided across all concentrations in experiment 3 (Fig. 5). Alternatively, the food ingested prior to the trails may have had varying nutritional qualities, which would increase or decrease the elephants’ toxin thresholds for monoterpenes and influence whether they considered the physiological repercussions of the monoterpenes (Illius and Jessop 1996). This could potentially explain why α-pinene was the only monoterpene that the elephants did not avoid at 30% concentration (Fig. 3). Previous studies have found that α-pinene concentration is negatively correlated with food consumption by goats and deer (Vourc’h et al. 2002; Adams et al. 2013). However, when supplied with increased dietary protein, goats and sheep do not respond to changes in α-pinene levels in one-seed juniper leaves (Utsumi et al. 2009). Alternatively, α-pinene consumption may even benefit herbivores by enhancing fermentation (Broudiscou et al. 2007). Given that the effects of individual monoterpene consumption on elephant physiology remain unknown, it is not yet possible to definitively ascertain whether monoterpenes act as a direct defence against elephant herbivory (Boyle et al. 1999; Estell 2010; Camp et al. 2015).

Another explanation for the elephants’ avoidance of monoterpene odours may be that monoterpenes act as an indirect defence against elephant herbivory. In particular, the elephants may associate certain monoterpene odours with the avoided plants in which they occur (Lawler et al. 1998; Moore et al. 2004; Massei et al. 2007; Dicke and Baldwin 2010). For example, Navon et al. (2020) found that the primary monoterpene hydrocarbons present in *Pistacia lentiscus* influenced the dietary choices of goats (*Capra hircus*). Specifically, goats preferred *P. lentiscus* shrubs with chemotypes dominated by limonene compared to chemotypes dominated by α-pinene. However, both chemotypes have the same nutritional value, and the total concentration of the monoterpenes within these plants were likely too low to elicit toxic effects (Navon et al. 2020). As such, α-pinene may serve as a signature VOC that goats associate with other plant species that have low nutritional quality (Navon et al. 2020). Similarly, the mean relative abundances of monoterpenes in the vegetation of the elephants’ habitat confirm that most of the monoterpenes used in this study are more abundant in avoided plants than preferred plants, with linalool being the exception (Schmitt et al. 2020c).

As an indirect defence mechanism, not all monoterpene odours would necessarily be associated with negative post-ingestive feedback. In experiment 3, the elephants did not avoid linalool and sabinene odours at 5% and 10% concentrations (1.7 ± 0.04% DW and 3.4 ± 0.09% DW, respectively; Fig. 5). At these added levels, the observed total content of linalool and sabinene within the *E. crispa* branch (5 g dry weight) may be lower than what is found in the plants that the elephants avoid consuming and, thus, not associated with the post-ingestive feedback of avoided foods (e.g., fatigue from malnourishment, malaise from other PSMs). However, the natural range for monoterpenes as a percentage of dry weight in the vegetation consumed by the elephants is unknown, which limits our interpretation of these results.

Monoterpenes have also previously been observed to act as foraging cues for herbivores like woodrats (*Neotoma stephensi*), which forage on juniper trees (*Juniperus osteosperma*) with higher concentrations of ρ-cymene (Skopec et al. 2019), swamp wallabies (*Wallabia bicolor*), which feed more extensively of foods containing elevated levels of 1,8-cineole (Bedoya-Pérez et al. 2014), and koalas (*Phascolarctos cinereus*), which prefer *Eucalyptus* foliage with higher proportions of monoterpenes (Hume and Esson 1993). This could potentially explain why the elephants did not avoid all monoterpene odours. For example, α-pinene has been positively correlated with woodrat (*Neotoma lepida*) herbivory, with browsed *J. osteosperma* trees containing up to four times more α-pinene than non-browsed *J. osteosperma* trees (Adams et al. 2016). Moreover, *J. osteosperma* trees with high α-pinene content also had lower total amounts of oxygenated monoterpenes (Markó et al. 2008; Adams et al. 2016), which previous studies have found to generally be more bioactive and thus a greater deterrent than hydrocarbon monoterpenes (Schwartz et al. 1980; Vourc’h et al. 2002). As such, Adams et al. (2016) proposed that α-pinene could serve as a foraging cue for woodrats that indicates low levels of oxygenated monoterpenes. Ultimately, the reason why the elephants did not avoid the odour of α-pinene in our study remains unclear. Future research on whether elephants utilise monoterpene odours like α-pinene as foraging cues could potentially highlight a far more nuanced role that these compounds play in elephant foraging decisions.

The elephants’ tendency to select the control bucket could also be explained by mechanisms that do not necessitate a learnt association between the pre-ingestive cues of monoterpene odours and negative post-ingestive feedback. Instead, the elephants may have inherited conditioned food aversions through their mothers’ milk (i.e., pre-weaning) or by observing the foraging behaviours of other herd members while being weaned (Thorhallsdottir et al. 1990; Biquand and Biquand-Guyot 1992; Myers and Sclafani 2006). Pre-weaning food preference conditioning can influence mammalian diet choice by increasing their exposure to compounds that alter the flavour of milk, which could increase their acceptability of foods containing these compounds (Myers and Sclafani 2006). The viability of inherited food aversions through mother’s milk as an explanation for our results remains speculative, seeing as only two of the elephants are siblings (Mussina and Nuanedi). However, herd foraging behaviour and consequential social transmission of food preferences and aversions may have contributed to the dietary choice patterns in the elephants, seeing as four of the five elephants were originally obtained from the same habitat (Greater Kuduland Safaris, Soutpansberg, Limpopo, South Africa; 22°36′15.0″S, 30°10′25.2″E), with the exception being Chova (Farm Grootgeluk, Soutpansberg, Limpopo, South Africa; 24°31′0.1″S, 28°43′0.1″E).

Alternatively, the monoterpene odours within the 2 mL solutions may have masked the odour of *E. crispa* at high (i.e., 20% and 30%) concentrations, which would hamper the elephants’ ability to detect the potential food reward within the buckets containing the monoterpene solution. Consequently, the elephants’ preference for the control buckets in experiment 1 and 3 could instead be explained by their search for food rather than their avoidance of individual monoterpene odours at 20% and 30% concentration (Figs. 3, 5). Similar odour masking effects have been observed to decrease the consumption of pelleted food by swamp wallabies (*Wallabia bicolor*) when masked by the odour of the monoterpene, 1,8-cineole (Bedoya-Pérez et al. 2014). Given that elephants can distinguish between preferred and avoided plants via olfactory cues, even when the plants are hidden in a novel set of added odour cues (McArthur et al. 2019), it is likely that, at the lowest tested concentrations of the 2 mL monoterpene solutions (i.e., 5% and 10% concentration), the elephants could identify the food reward. If so, the elephants' avoidance of the individual monoterpene odours at low concentrations in experiment 3 was likely not due to odour-masking, but previously learnt or inherited aversions (Provenza 1995, 1996; Myers and Sclafani 2006). In particular, learnt aversions would be reinforced when the elephants went out to forage in their environment where the usage of pre-ingestive odour cues are necessary to identify plants with low nutritional quality and/or avoid the negative effects of plant chemical defences (Provenza 1995, 1996; Navon et al. 2020). In experiment 2, both buckets contained monoterpene solutions at 30% concentration, which would both likely mask the odour of *E. crispa*. Yet the elephants still showed clear preference/avoidance towards the individual monoterpene odours in certain choice combinations (Fig. 4). Whether high concentrations of monoterpenes deter elephant through odour masking remains unknown, but our results suggest that this is not the sole explanation for the elephants’ avoidance towards individual monoterpene odours.

Another possibility is that, during the training phase, the elephants learned to select the bucket containing the unaltered odour of *E. crispa* when compared to a bucket with any other odour. This would mean that the preference that the elephants show towards the control buckets would be better explained by their preference for the familiar odour of *E. crispa* and not their learnt or inherited aversions to monoterpenes (Provenza 1995, 1996). However, during training, the elephants did not receive any rewards for their decisions other than the contents of the chosen bucket, which was either a plant that they are sometimes willing to consume (*E. crispa*, acceptability index = 0.28) or a plant that they never consume (*O. europaea* subsp. africana, acceptability index = 0.00; Schmitt 2017, Schmitt et al. 2018). Indeed, the elephants did not always consume the *E. crispa* after choosing the bucket that contained it, which implies that their choices were not only driven by their relative preference for *E. crispa*, but also their avoidance of *O. europaea* subsp. africana. Because neither of the choices provided the elephants with a highly preferable food reward, the training instilled an understanding of “you-get-what-you-choose” as opposed to seeking out a specific odour (i.e., the unaltered odour of *E. crispa*). The elephants would not show random selection towards α-pinene at 30% concentration or linalool at 5% and 10% concentration if their choices were driven towards the unaltered odour of *E. crispa*, because neither of these monoterpenes naturally occur within the plant’s monoterpene profile (Figs. 3, 5; Schmitt et al. 2020c). As such, we conclude that the choices made by the elephants during experiments 1 and 3 indicated whether the odour of the individual monoterpenes decreased their preference for *E. crispa* rather than its recognisability (Figs. 3, 5). In experiment 2, the odour of the individual monoterpenes likely altered the odour of *E. crispa* to similar degrees, yet the elephants showed clear preference towards certain individual monoterpene odours when compared to other monoterpenes (Fig. 4). Furthermore, the elephants did not consistently choose the buckets containing the odours of monoterpenes that were already present in *E. crispa* (i.e., β-pinene, limonene, and γ-terpinene; Schmitt et al. 2020c) when compared to the odours of monoterpenes that were not present in *E. crispa* (Fig. 4). These results further imply that the elephants did not specifically target the buckets containing odours that resembled the odour profile of unaltered *E. crispa*, but rather based their decisions on which individual monoterpene odour was more of a deterrent.

We used *E. crispa* as a food reward because, as previously mentioned, it is a principal plant that is frequently encountered and consumed, but not preferred, by the elephants (Schmitt et al. 2020c). As a principal plant, elephants rely on *E. crispa* for survival, and its non-preference implies that a shift in observed monoterpene content would more accurately reflect on elephant diet choice than if a preferred, highly nutritious food was used. Furthermore, the selective pressure for the elephants to disregard the increase in monoterpene content (i.e., learning extinction) of the *E. crispa* food reward during the experiments would likely not be enough to alter their overall diet choices, seeing as the food offered to the elephants during a full day of experimentation (~ 75 gDW) constituted a very small portion of their daily intake (Rees 1982; Ruggiero 1992). Since the elephants did not ingest the added monoterpenes, the consumption of *E. crispa* in this study did not lead to increased post-ingestive costs. Yet, at certain concentrations, the elephants still avoided the buckets with the odour of the 2 mL individual monoterpene solutions (Figs. 3, 5), despite the food rewards for both options (control and individual monoterpenes) yielding similar post-ingestive feedback. The elephants also displayed clear preference for certain individual monoterpene odours over others (Fig. 4). As such, we believe that our results further highlight the key role that olfactory cues play in elephant diet choice.

Similar smell–smell–choose experimental model have previously been utilised to investigate the elephant diet choice and their ability to detect preferred food sources via olfaction (Schmitt et al. 2018; Nevo et al. 2020). However, unlike Schmitt et al (2018) and Nevo et al. (2020), our study aimed to determine whether elephant dietary preference for a food item could be influenced by only modifying the odour of the food via the addition of monoterpene odours. Thus, the efficacy of the smell–smell–choose experimental model in our study was underpinned by the elephants’ proven ability to identify a less preferred food source and to adjust their dietary choices accordingly. Ultimately, although the underlying mechanisms that drove elephant avoidance of monoterpene odours in our study (i.e., learnt associations, inherited aversions, odour-masking) are unknown, the pattern of elephants avoiding individual monoterpene odours, and this avoidance differing among individual monoterpenes and at varying concentrations, remains clear.

## Data Availability

The data for this work was deposited into the Institutional Repository of the University of Pretoria at https://doi.org/10.25403/UPresearchdata.20226015.v1 and https://doi.org/10.25403/UPresearchdata.20461833.v1
